# Stability of Tandemly Repetitive Subelement PCR Patterns in *Trichophyton rubrum* over Serial Passaging and with Respect to Drug Pressure

**DOI:** 10.1007/s11046-012-9565-4

**Published:** 2012-07-20

**Authors:** Anita Hryncewicz-Gwóźdź, Tomasz Jagielski, Katarzyna Kalinowska, Dagmara Baczyńska, Ewa Plomer-Niezgoda, Jacek Bielecki

**Affiliations:** 1 Department of Dermatology, Venereology and Allergology, Wrocław Medical University, Wrocław, Poland; 2 Department of Applied Microbiology, Institute of Microbiology, Faculty of Biology, University of Warsaw, I. Miecznikowa 1, 02-096 Warsaw, Poland; 3 Molecular Techniques Unit, Department of Forensic Medicine, Wrocław Medical University, Wrocław, Poland

**Keywords:** *Trichophyton rubrum*, TRS typing, Genotype stability

## Abstract

*Trichophyton rubrum* is the most significant agent of dermatomycoses worldwide, primarily causing *tinea pedis* and *tinea unguium*. PCR analysis of tandemly repetitive subelements (TRS) within the rDNA nontranscribed spacer region is a major tool for molecular typing of *T. rubrum*. The aim of this study was to investigate the stability of TRS PCR patterns by analyzing isogenic strains of *T. rubrum*. Twenty-seven groups of isogenic *T. rubrum* strains were examined, each composed of an original clinical isolate and its 3 subcultures, maintained on a drug-free medium, a medium containing fluconazole and itraconazole. TRS typing was performed for the original strains and their subcultures grown after 12 passages, at 4-week intervals, on respective media. To add more objectivity to the results, TRS typing for each of the isogenic strain was performed three times, using DNA isolated from three different colonies. Among 27 groups of isogenic strains, all but one were exclusively composed of strains with identical TRS-1 and TRS-2 PCR patterns. In one group, 3 isolates from the last, twelfth passage had identical TRS-1 PCR profiles (type 1), yet different TRS-2 PCR profiles, as compared with the original strain (type I vs. type II). The mechanism underlying the genotype switch was a deletion of a single repeat unit in the TRS-2 locus, as evidenced by sequence analysis. In the interpretation of TRS typing results, microevolutionary events need to be taken into account, urging drawing epidemiological conclusions with caution and in conjunction with other genotyping data and traditional contact tracing information.

## Introduction

*Trichophyton rubrum*, an obligatory anthropophilic dermatophyte species, is the most significant agent of dermatomycoses worldwide, primarily causing *tinea pedis* (athlete’s foot) and *tinea unguium* (onychomycosis) [1]. In Poland, *T. rubrum* ranks the first among the causative agents of both these conditions, as well as of all other superficial skin infections reported, with the frequency of isolation exceeding 45 % [2].

With the advent of molecular typing methods, mapping of polymorphisms between individual genomes has become a major experimental tool to explore the epidemiology of many fungal infections.

*Trichophyton rubrum* has long been considered an extensively clonal species, since several anonymous DNA markers failed to reveal any substantial interstrain genomic variation [3]. The genetic uniformity of *T. rubrum*, clearly contrasting with its high phenotypic variability, supported the scenario that the species is a product of a very recent evolutionary radiation [4].

Recently, however, some genetic variation among *T. rubrum* strains has been demonstrated by using the amplification of tandemly repetitive subelements (TRS) within the rDNA nontranscribed spacer (NTS) region [5], randomly amplified polymorphic DNA (RAPD) analysis [6–8], multilocus microsatellite typing (MLMT) [9, 10], and PCR melting profile (PCR-MP) typing [11]. It is to mention, however, that none of these methods have yet gained a status of a gold standard in *T. rubrum* genotyping. This is because each method suffers from certain limitations. For example, the TRS typing targets only a single locus (NTS) that accounts for a very fragmentary part of the *T. rubrum* genome [5]. On the other hand, the RAPD method acts at the whole-genome level, but often lacks reproducibility and inter-laboratory comparability [7]. Finally, most of the methods currently used for *T. rubrum* typing are helpless in providing answers to key questions regarding the epidemiology of dermatophyte infections, such as whether multiple lesions are caused by the same or different strains, whether recurrent infections are due to the involvement of a new strain or reactivation of an old one, or are there any associations between strain types and their geographical origin or clinical picture of the patients [5, 10].

So far, the data concerning the stability of the aforesaid markers are very scanty. In this study, the stability of *T. rubrum* TRS typing patterns was investigated by analyzing groups of isogenic strains that are composed of an original clinical isolate and its subcultures.

## Materials and Methods

### Isolates: Cultivation and Passaging

The study included 27 isolates of *T. rubrum* recovered from 26 *tinea* patients (two isolates derived from two different sites on a single patient) from Lower Silesia, Poland, between May 2006 and April 2007. Primary isolation and species identification were performed essentially as described previously [12]. The strains were maintained on Sabouraud dextrose agar (SDA) slopes at 4 °C for 1 year. Each isolate was subcultured at least twice on SDA medium before passaging, to ensure its purity and optimal growth.

To investigate the genotype stability, 27 groups of isogenic strains of *T. rubrum* were established. Briefly, mycelium from a single colony of each of the 27 clinical strains served as an inoculum for setting up three parallel subcultures, that is, maintained on a drug-free medium (1), a medium supplemented with either fluconazole (2) or itraconazole (3), at a concentration of 2 μg/mL and 0.125 μg/mL, respectively. (The chosen drug concentrations were the highest, at which growth of all the strains occurred, as evidenced by microdilution susceptibility testing, performed following the CLSI M28-A reference method [13]). The strains were passaged 12 times at 4-week intervals on respective media, and with every subsequent passage, the same single colony served as a source of a new culture on all three types of media.

### DNA Isolation

For extraction of fungal DNA, the mini-preparation procedure of Liu et al. [14] was used, with some modifications, as described elsewhere [12].

### PCR Amplification of the TRS Loci

Amplification of TRS-1 and TRS-2 loci was performed, as previously described [12]. Briefly, primers TrNTSF-2 (5′-ACC GTA TTA AGC TAG CGC TGC-3′) and TrNTSR-4 (5′-TGC CAC TTC GAT TAG GAG GC-3′) were used to amplify TRS-1, and primers TrNTSR-1 (5′-CTC AGT CGA ACC GTG AGG C-3′) and TrNTSC-1 (5′-CGA GAC CAC GTG ATA CAT GCG-3′) to amplify TRS-2. PCR mixtures were prepared by using the QIAGEN multiplex PCR kit (QIAGEN, Germany) in a total volume of 25 μL, containing 2× QIAGEN multiplex PCR master mix (final conc. 1×), 1 μL of DNA, and 0.2 μM of each of the two primers. Thermocycling conditions were as follows: 1 cycle of 15 min at 95 °C, 30 cycles of 30 s at 94 °C, 30 s at either 58 °C (TRS-1) or 55 °C (TRS-2), and 3 min (TRS-1) or 2 min (TRS-2) at 72 °C, and a final extension for 10 min at 72 °C.

The PCR products were separated by electrophoresis in 2 % agarose gels, visualized by staining with ethidium bromide and photographed in UV light.

The TRS typing was performed for each twelfth generation culture of each original *T. rubrum* strain, as well as for the original strain itself. Furthermore, to add more objectivity to the results, TRS typing for each of the isogenic strain was performed three times, using DNA isolated from three different colonies. Hence, a total of 324 PCRs were performed (12 PCRs per each of the 27 groups of isogenic *T. rubrum* isolates).

## Results and Discussion

The results of the PCR profiling at two loci, TRS-1 and TRS-2, for all the *T. rubrum* isolates under the study, are shown in Table 1.

**Table 1:** Results of TRS PCR typing of 27 *T. rubrum* clinical isolates and their 12th generation subcultures on three types of media

	Strain no.	Primary genotype		TRS-1 type after 12 passages on medium containing			TRS-2 type after 12 passages on medium containing		
		TRS-1	TRS-2	–^a^	FTZ^b^	ITZ^c^	–	FTZ	ITZ
1.	860/07	1	II	1	1	1	II	II	II
2.	274/07	1	II	1	1	1	II	II	II
3.	171/07	1	II	1	1	1	II	II	II
4.	300/07	1	II	1	1	1	II	II	II
5.	872/07	1	II	1	1	1	I	I	I
6.	1725/06	1	II	1	1	1	II	II	II
7.	934/07	1	II	1	1	1	II	II	II
8.	857/07	1	II	1	1	1	II	II	II
9.	799/07	1	II	1	1	1	II	II	II
10.	908/07	1	III	1	1	1	III	III	III
11.	718/07	1	II	1	1	1	II	II	II
12.^d^	987/07	1	II	1	1	1	II	II	II
13.	312/07	1	I	1	1	1	I	I	I
14.	390/07	1	II	1	1	1	II	II	II
15.	609/07	1	II	1	1	1	II	II	II
16.	866/07	1	II	1	1	1	II	II	II
17.	204/07	1	II	1	1	1	II	II	II
18.	781/07	1	II	1	1	1	II	II	II
19.	1766/06	1	II	1	1	1	II	II	II
20.^d^	1775/06	1	II	1	1	1	II	II	II
21.	265/07	1	II	1	1	1	II	II	II
22.	851/07	1	II	1	1	1	II	II	II
23.	59/07	2	II	2	2	2	II	II	II
24.	980/07	2	II	2	2	2	II	II	II
25.	1015/07	3	II	3	3	3	II	II	II
26.	999/07	1	II	1	1	1	II	II	II
27.	647/07	1	II	1	1	1	II	II	II

^a^No drug

^b^Fluconazole

^c^Itraconazole

^d^Strains isolated from the same patient

**Fig. 1:**
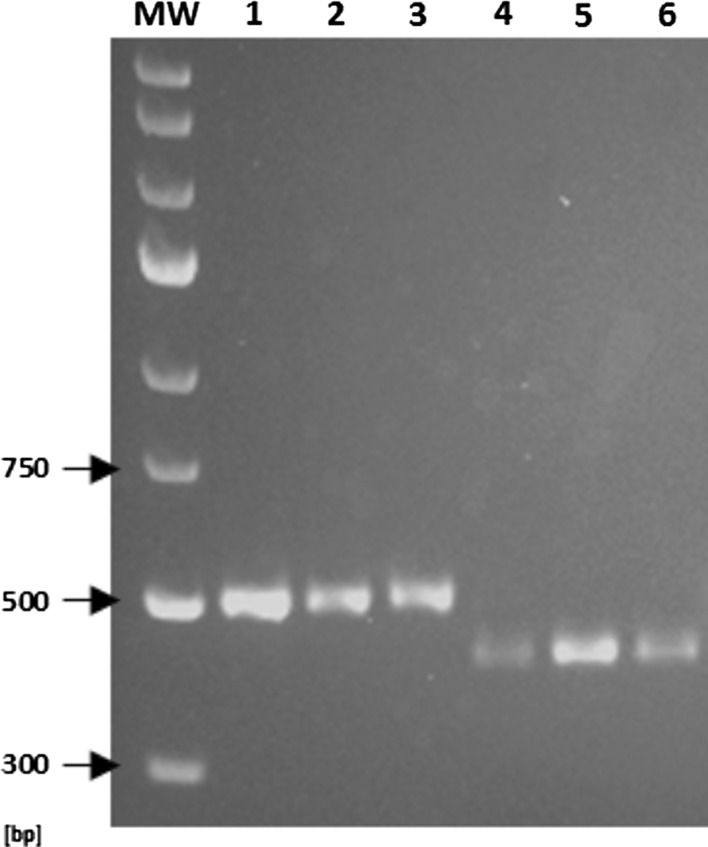
A switch in the TRS-2 PCR type in a *T. rubrum* strain no. 872/07. Lanes: MW, molecular weight marker (GeneRuler™ Express DNA Ladder, Fermentas); 1–3, three random colonies of the original clinical isolate; 4–6, twelfth generation subcultures of the original isolate growing on a medium with no drug (4), with fluconazole (5) or itraconazole (6)

Among 27 groups of isogenic isolates (i.e., one original clinical strain plus 3 progeny isolates), all but one were exclusively composed of isolates with identical TRS-1 and TRS-2 PCR patterns. In one group, three isolates (i.e., from all three types of culture media) from the last, twelfth passage had identical TRS-1 PCR profiles (type 1), yet different TRS-2 PCR profiles, as compared with the original strain (type I *vs.* type II) (Fig. 1). Hence, a combined TRS genotype was 1-II for the original strain and 1-I for its 3 subcultures, grown after twelve rounds of passaging on three different media. The typing results for the three colonies of each of the strain were always consistent. The observed genotypic variation was not correlated with any detectable phenotypic variation.

This study is, to the best of the authors’ knowledge, the first to report a change in the NTS genotype in *T. rubrum* isogenic strains (subculture derived in the laboratory). Changes in the *T. rubrum* DNA patterns, specifically the rDNA restriction fragment length polymorphism (RFLP) patterns, have been observed among serial isolates recovered from the same or different anatomical sites on the same patients over at least a 1-year period [4]. Distinct TRS PCR types have also been demonstrated for individual colonies of the same specimen from patients with onychomycosis [15]. Apart from the study of Jackson et al. [6], where stability of TRS PCR types has been evidenced for a *T. rubrum* reference strain, cultured in vitro over a 2-year period, the only study that has attempted to explore the stability of *T. rubrum* genotypes in isogenic strains revealed no changes in both rDNA RFLP and arbitrarily primed (AP) PCR patterns [16]. In the study of Jackson et al., the precise methodology used for subculturing and colony sampling remains somewhat obscure. Otherwise, Guoling et al. [16] analyzed only 11 *T. rubrum* strains and performed only 4 passages, with 4-week intervals, which might have been a possible reason for not finding any altered genotypes.

**Fig. 2:**
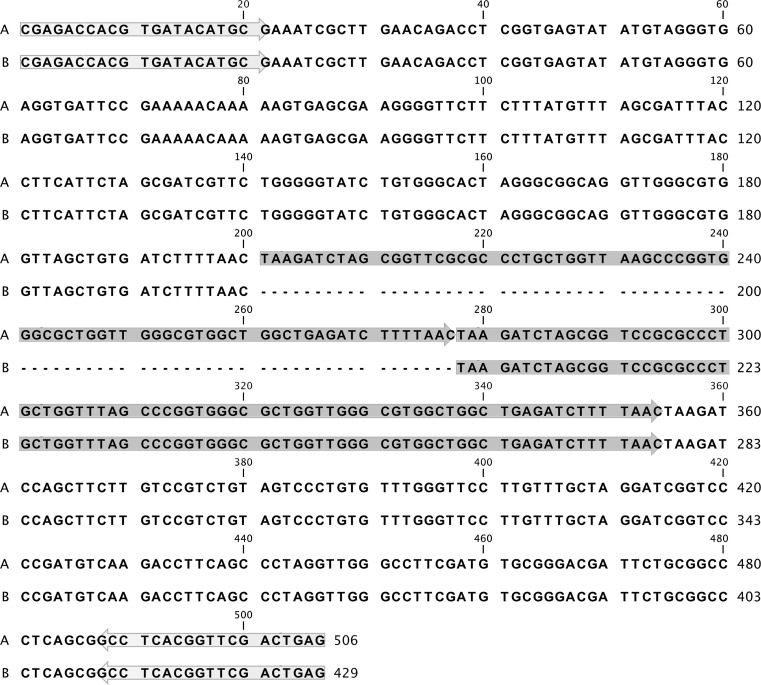
Alignment of the TRS-2 element sequences derived from the original clinical isolate no. 872 (*A*) and one of its subcultures, grown after 12 passages (*B*). The TRS-2 repeat units (77 bp in length) are *boxed in gray*. The lack of a single copy of the TRS-2 element in the progeny isolate of the strain no. 872 is marked with *dots*. The priming sites are *boxed in light gray*. The alignment was performed with the CLC Main Workbench 6.0 (CLC bio, Aarhus, Denmark)

Whereas the presence of different genotypes in a nail of a single patient may reflect a multiple infection, co-inhabitation of multiple strains, or microevolutionary events [4], only the latter explanation can account for the observation from this report. Indeed, further analysis of the original strain and its three filial subcultures bearing a TRS-2 profile change, based upon sequencing of the TRS-2 locus, revealed a deletion of a single repeat unit (Fig. 2). This finding corroborates the previously hypothesized mechanism underlying variations in the copy number of the NTS subrepeats. Interestingly, this mechanism seems to be independent of culture conditions, such as the presence of a drug in culture medium. Although the study left some questions unanswered, such as how many passages, exactly, were needed to produce a genetic variant of the original strain or whether prolonged passaging would yield more altered genotypes, detection of a clear genotype switch in one strain over a 1-year period is enough to suggest that the rate of microevolution in the *T. rubrum* NTS region, or the so-called molecular clock of this particular genetic marker, is rather fast.

Finally, although a deletion that resulted in a genotype switch occurred in isogenic strains, it is likely that similar deletion events take place in vivo, perhaps only governed by different molecular clocks. This in turn may have important implications for the epidemiological investigation of *T. rubrum* infections. A link between an original strain and its genetic variant (i.e., strain that underwent, in the same patient, a microevolutionary change) would have been missed. Therefore, interpretation of TRS typing results should be made with caution and in conjunction with other genotyping data (e.g., MLMT) and traditional contact tracing information.
